# Reversible diabetes mellitus induced by use of, and improved after discontinuation of, the antiretroviral medication zidovudine: a case report

**DOI:** 10.1186/s13256-017-1326-z

**Published:** 2017-06-14

**Authors:** Kentaro Iwata, Wataru Ogawa

**Affiliations:** 1 Division of Infectious Diseases, Department of Microbiology and Infectious Diseases, Kobe University Graduate School of Medicine, 7-5-2 Kusunokicho, Chuo-ku, Kobe, Hyogo 650-0017 Japan; 2 Division of Diabetes and Endocrinology, Department of Internal Medicine, Kobe University Graduate School of Medicine, Kobe, Japan

**Keywords:** HIV infection, Diabetes mellitus, Zidovudine

## Abstract

**Background:**

With the advent of effective antiretroviral therapy, the care of patients with human immunodeficiency virus infection became more like that of other chronic diseases. Diabetes mellitus can also occur as one of the chronic illnesses affecting patients with human immunodeficiency virus infection. We report a case of newly developed diabetes mellitus in a patient with human immunodeficiency virus infection, most likely caused by the nucleoside analogue zidovudine, and its improvement after discontinuation of zidovudine.

**Case presentation:**

A Chinese man in his 30s visited our outpatient clinic for routine follow-up of human immunodeficiency virus infection. Blood tests showed hyperglycemia with a glucose level of 31.8 mmol/L and hemoglobin A1c of 8.5%. He was diagnosed with diabetes mellitus and treated with oral diabetic medications. The use of zidovudine was suspected as the cause of his diabetes, and it was replaced by other antiretroviral medication. His hyperglycemia improved, and he now no longer requires diabetic medications.

**Conclusions:**

Diabetes mellitus can develop with the use of antiretroviral medications, but its occurrence associated with use of zidovudine is quite rare. Healthcare personnel should be aware of this rare, yet important, side effect.

## Background

With the advent of effective antiretroviral therapy (ART; formerly known as *highly active antiretroviral therapy*, or *HAART*) in late 1990s, the prognosis of patients with human immunodeficiency virus (HIV) infection improved significantly. The patients live longer, and as a result they face more health problems associated with aging [1]. Although HIV infection *per se* is not known to be associated with increased incidence of diabetes mellitus, some antiretroviral agents, such as protease inhibitors, may increase the risk of developing diabetes [2]. We report a rare case of a patient with diabetes mellitus, most likely caused by zidovudine, a classical nucleoside reverse transcriptase inhibitor, which improved by discontinuing the medication.

## Case presentation

A Chinese man in his 30s visited our outpatient clinic for routine follow-up of HIV infection. He had become infected with HIV about 10 years earlier and had been followed at our outpatient clinic since 7 years prior to the current presentation. He had never had hyperglycemia during follow-up. Upon arrival, he stated that he was in his usual state of health and denied any symptoms such as polydipsia or polyuria, except for weight loss, but he did not recall the precise amount of weight loss or its duration. He stated that he was an occasional binge drinker of alcohol but denied any alcohol intake since 1 month prior to the current presentation. His height was 162 cm, and his weight was 42.8 kg. His physical examination was unremarkable, and he did not have any bodily habitus to suggest lipodystrophy. His past medical history, besides HIV infection, included depression, multiple alcohol binge-drinking periods with repeated episodes of acute pancreatitis, hypertriglyceridemia, and two episodes of amoebic liver abscess. His medications included Combivir® (zidovudine and lamivudine; ViiV Healthcare, Brentford, UK), raltegravir, and fenofibrate. He was seen by a psychiatrist and had received sertraline, levomepromazine, flunitrazepam, and quazepam, but he had discontinued all medications approximately 1 month prior to the current presentation after discussion with his psychiatrist. He denied tobacco use, illicit drug use, or use of any supplements. He had been taking Combivir® (zidovudine and lamivudine) and KALETRA® (lopinavir/ritonavir; AbbVie, Chicago, IL, USA) for several years, but KALETRA® was switched to atazanavir/ritonavir because of an interaction with psychiatric medications 6 years ago. They were again changed to raltegravir 4 years ago because of hypertriglyceridemia. With ART consisting of Combivir® and raltegravir, the patient’s HIV infection had been stable, with a latest CD4^+^ T lymphocyte count being 1065/mm^3^ and an undetectable viral load (Table 1).

**Table 1:** Laboratory results of the patient

Variables	Reference ranges, adults	On day of visit	Blood test results 2 months after discontinuing diabetes medications
White cell count, per mm^3^	4000–8500	13,100	6400
Hemoglobin, g/dl	13.6–17	12.9	14.6
Platelet, per mm^3^	130,000–300,000	381,000	341,000
Sodium, mmol/L	137–146	130	136
Potassium, mmol/L	3.5–4.7	4.3	3.5
Chloride, mmol/L	99–109	96	110
Calcium, mg/dl	8.8–10.1	10.1	Not measured
Phosphorus, mg/dl	2.4–4.5	4.4	Not measured
Albumin, g/dl	4.1–5	4.6	4.9
Aspartate aminotransferase, U/L	13–31	36	25
Alanine aminotransferase, U/L	8–34	106	37
Total bilirubin, mg/dl	0.3–1	0.5	0.5
Blood urea nitrogen, mg/dl	9–22	14.5	12.1
Creatinine, mg/dl	0.5–1.3	0.69	0.79
Glucose, nonfasting, mmol/L	4.0–6.0	31.8	7.5
Hemoglobin A1c, %		8.5	6.0
Blood gas, arterial, room air			
pH	7.38–7.46	7.404	
Partial pressure of oxygen, mmHg	74–108	115.0	
Partial pressure of carbon dioxide, mmHg	32–46	36.8	
Bicarbonate, mmol/L	21–29	22.5	
Anion gap, mmol/L	10–20	11.5	
CD4^+^ T-lymphocyte count, per mm^3^		1065	865
HIV RNA, copies/ml		Undetectable	Undetectable

*AST* Aspartate aminotransferase, *ALT* Alanine aminotransferase, *BUN* Blood Urea Nitrogen, *PaO2* Partial pressure of oxygen, *PaCO2* Partial pressure of carbon dioxide, *HCO3* Bicarbonate, *HIV* human immunodeficiency virus, *RNA* ribonucleic acid

Blood tests done on the day of the current presentation showed elevated glucose of 31.8 mmol/L, sodium 130 mmol/L, and potassium 4.3 mmol/L, with mildly elevated liver transaminases. The patient’s hemoglobin A1c was 8.5%. His arterial blood gas analysis on room air showed a pH of 7.404, partial pressure of oxygen of 115 mmHg, partial pressure of carbon dioxide of 36.8 mmHg, bicarbonate of 22.5 mmol/L, and an anion gap of 11.5 mmol/L (Table 1).

Because of the patient’s hyperglycemia without acidosis, we presumptively diagnosed that he had newly developed diabetes mellitus and considered the possibility of insulin depletion as a result of repeated acute pancreatitis. We then referred him to our endocrinology clinic. Additional blood tests were ordered, and it turned out that his blood C-peptide level was 2.69 ng/ml (normal range 0.69–2.45 ng/ml), his blood insulin level was 15 μU/ml (normal range 5–30 μU/ml), and his glutamic acid decarboxylase antibody level was 0.6 U/ml (normal range 0–1.49 U/ml). Unlike our initial assessment, we then judged him to have diabetes mellitus with preserved insulin secretion from the pancreas, and he was prescribed glimepiride 1 mg once daily. Sitagliptin 50 mg once daily and metformin 250 mg twice daily were added later, and his hyperglycemia normalized with a decreasing hemoglobin A1c level. A computed tomographic scan of the patient’s abdomen with contrast did not show any evidence of chronic pancreatitis, but it showed sporadic low-density areas in the liver, suggesting partial fatty liver.

By this time, we began to suspect the antiretroviral medications, particularly zidovudine, as the cause of the patient’s diabetes. Combivir® was switched to TRUVADA® (tenofovir disoproxil and emtricitabine; Gilead Sciences, Foster City, CA, USA) 4 months after the detection of diabetes mellitus. His hyperglycemia further improved, and his medications for diabetes were decreased. Seven months after the patient’s initial presentation, all diabetes medications were stopped. He remained stable after discontinuing all diabetic medications, with a fasting glucose level of 7.5 mmol/L and a hemoglobin A1C of 6.0% (9 months after the initial visit) (Table 1).

## Discussion

The patient developed diabetes mellitus with relatively preserved insulin secretion while on ART, and the discontinuation of zidovudine improved his hyperglycemia to a level where he did not require diabetes medications. Because lamivudine and emtricitabine are structurally very similar [3], switching from lamivudine to emtricitabine is unlikely to have contributed to improving his hyperglycemia. He had been receiving protease inhibitors (lopinavir/ritonavir, then atazanavir with ritonavir, both of which are known to cause diabetes in patients with HIV infection [4–7]), but they were discontinued years before the current presentation and are unlikely to have been the cause of his diabetes.

Old antiretroviral medications such as zalcitabine, didanosine, stavudine, and medications for treating opportunistic infections, such as pentamidine, can cause pancreatitis and may lead to the development of diabetes [8–11]. Although rare, cohort studies showed that the use of zidovudine was associated with increased risk of developing diabetes [12–14], and it was an independent risk factor after adjusting for lipodystrophy [12].

The pathogenesis of diabetes caused by zidovudine remains unknown, but it may be associated with lipodystrophy or mitochondrial toxicity [12, 15]. Although our patient did not have the typical bodily habitus of lipodystrophy, some could suspect that his weight loss could have been a reflection of lipoatrophy caused by zidovudine, which could have contributed to the development of diabetes. However, the patient’s weight and appearance remained unchanged 5 months after stopping zidovudine. Replacement of the causative thymidine analogue usually leads to improvement of lipoatrophy [16]. He had partial fatty liver, which is a common finding of HIV-related lipoatrophy, but his history of binge alcohol intake also might have been the cause [17]. Therefore, it is more plausible to consider that his diabetes was caused by the direct effect of zidovudine, not secondary to lipodystrophy syndrome.

The management of diabetes in patients with HIV infection is basically the same as that for patients without HIV infection, but the discontinuation of ART medications likely to have caused diabetes is recommended [18]. Even though population-based studies suggested an association between the use of zidovudine and diabetes, we were not able to find any case report or series demonstrating the development of diabetes after the use of zidovudine (and improvement after its discontinuation).

One might consider that the discontinuation of medications for depression might have affected the pharmacokinetics or pharmacodynamics of zidovudine. We were not able to find any interaction between zidovudine and these medications, but this possibility has to be kept in mind because some drug interactions could remain unknown [19].

## Conclusions

We encountered a case of new-onset diabetes in a patient with HIV infection in whom the discontinuation of zidovudine improved glycemic control. The rare occurrence of diabetes due to the use of zidovudine would not necessarily preclude the use of this medication, but one should be aware of this side effect, particularly in patients who already have or have developed diabetes while receiving zidovudine.
